# Protocol of a case-control longitudinal study (fraXity) assessing frailty and complexity among Swiss home service recipients using interRAI-HC assessments

**DOI:** 10.1186/s12877-019-1230-z

**Published:** 2019-08-05

**Authors:** Catherine Ludwig, Catherine Busnel

**Affiliations:** 1 Geneva School of Health Sciences, HES-SO, University of Applied Sciences and Arts Western Switzerland, Avenue de Champel 47, 1206 Geneva, Switzerland; 2 Geneva Institution for Homecare and Assistance (imad), Avenue du Cardinal Mermillod 36, 1227 Carouge, Switzerland

**Keywords:** Frailty, Complexity, Standardized geriatric assessment, Home care, Nursing

## Abstract

**Background:**

The early screening of frail individuals and of patients with complex care needs are challenges that countries witnessing population aging face. Homecare nurses are actors of choice in meeting these challenges, yet they need means of identifying frail and complex patients in their routine practice. The fraXity study’s aim is to fill this gap by (1) proposing frailty and complexity computation algorithms derived from the interRAI-HC; (2) assessing the predictive validity of the proposed indices with respect to adverse health outcomes; and (3) identifying subgroups of the aged population for whom the early screening of frailty and complexity appears to be most relevant.

**Methods:**

The study will rely on a prospective observational case-control longitudinal study. Three samples of individuals aged 65 or older living in the community will be considered: recipients of formal home care (case 1), of formal home assistance (case 2) and individuals free of formal home services (controls). All participants will receive interRAI-HC assessments at three measurement occasions, separated by six-month intervals. Baseline assessments will serve to derive frailty and complexity scores. Follow-ups will serve to assess the predictive validity of the proposed indices and to estimate the intra-individual change in frailty and complexity. Group comparisons will serve to identify subgroups of the population for whom the screening of frailty and complexity appears to be the most relevant.

**Discussion:**

The expected results of the fraXity study are a) reliable computation algorithms for frailty and complexity scores derived from the interRAI-HC and b) clinical assessment protocols for use by homecare nurses. These outcomes should contribute to outfitting key actors of the health system with means of enhancing their part in a collective endeavor targeting the best care and quality of life for aged citizens.

**Trial registration:**

ClinicalTrials.gov, NCT03883425, registered on March 20, 2019.

## Background

### Scientific background

Like the populations of most industrialized countries [1], Switzerland’s population is aging [2]. In this context, noncommunicable chronic conditions and multimorbidities have tremendous costs for societies [3] and pose considerable challenges for health systems in terms of case and care management [4, 5]. Today, in forsaking the disease-centered approach [6], scholars and clinicians are calling for interprofessional integrated care [7] and the early identification of vulnerable individuals who are at risk for adverse health outcomes [4, 8]. In this paradigmatic turn, two challenges need to be taken up [1, 9, 10]. The first is the early screening of frail individuals who are at risk of functional decline, so as to immediately engage targeted preventive intervention [11]. The second challenge is the early identification of patients with complex care needs, so as to rapidly adjust care plans and avoid inappropriate treatments, incoherent interventions and unnecessary hospital admissions [12].

As a response to the needs of the aging population, Switzerland promotes “aging in place” [13] and fosters individualized integrated care [14] to reduce the barriers between hospitals and communities [15]. Yet, to be efficient, integrated care implies not only the coherent management of care delivery across settings and actors [16] but also the early identification of critical cases [9, 17] and situations [4, 12, 18, 19] that enhance the risk of undesirable health outcomes. In health systems characterized by an ambulatory switch, homecare nurses are actors of choice who actively take part in these endeavors [20].

In many countries [21, 22], including Switzerland [23], homecare nurses assess the health conditions and homecare needs of elders by using the Resident Assessment Instrument – Home Care (interRAI-HC, [24]) a reliable instrument dedicated to comprehensive geriatric assessment. The quality and nature of the information collected with the RAI-HC are rich enough to derive additional scores featuring relevant properties for clinical management [25]. Of particular interest to the fraXity study is recent and convincing evidence that frailty scores can be derived directly from RAI instruments, including the interRAI–Acute Care [26, 27], the interRAI–Home Care [28–31] and the Swiss RAI–Home Care [32]. Thus, strong working bases are available for developing estimates of frailty directly from instruments that homecare nurses routinely use. Such tools would serve as opportunities for them to systematically assess—and thus efficiently screen for—frailty among home service receivers.

Aside from frail patients, homecare professionals are increasingly confronted with patients who have compound, chronic and yet fluctuating clinical pictures [33] and who are at high risk of decompensation and hospital readmission [34, 35]. It is acknowledged today that efficient integrated care management and reactive coordination among actors can improve the quality of care, reduce health resource consumptions and improve a patient’s care experience and quality of life [36]. Provided with these benefits, clinicians and economists call today for the early identification of “high needs–high cost” patients, so as to provide immediate care adjustment, prevent potentially avoidable hospital admission and reduce costs for both the patients and the health system [36]. With respect to nursing practice, a multidimensional scale of complexity (COMID) was recently proposed [37] as a decision tool designated specifically for homecare nurses. The COMID items can potentially be documented from information previously gathered by means of a comprehensive geriatric assessment done with the RAI-HC. As for frailty, stirring evidence is available to develop multidimensional complexity screening tools that will give homecare nurses the opportunity to identify complex patients who deserve rapid care-plan adjustments so as to avoid undesirable health outcomes and unnecessary health expenditures.

### Objectives

The main purpose of the fraXity study is to develop frailty and complexity index computation algorithms based on the Canadian French version of the interRAI-HC [38]. The frailty index (FI) derivation relies on the available methodology [39]. This well-documented rationale [26–32], further supported by the operational definition proposed in the COMID [37], is used to derive the complexity index (CI). By relying on a prospective observational case-control longitudinal design, the study aims to assess the predictive validity of the proposed indices with respect to undesirable health outcomes (falls, emergency admissions, deaths) and health resource consumption (length of hospital stays, number of physician visits), monitored throughout the course of the study. Three measurement occasions will be used to estimate intra-individual change in frailty and complexity, as assessed with the proposed indices. Finally, by considering different subgroups of the aged population (see below), the study should provide estimates of frailty and complexity rates in a panel of the older population wider than the usual clinical population that interRAI instruments target. Overall, the fraXity study addresses four research questions. (1) Can frailty, respectively complexity, indices be derived from information collected during a comprehensive geriatric assessment done with the RAI-HC? (2) What is the predictive validity of frailty, respectively complexity, indices with respect to adverse health outcomes and health resource consumption? (3) What are the rates of frailty, respectively complexity, in various subsamples of the older population, and how do these rates change over time? (4) Which recommendations for best practices can be drawn from the findings to mount care strategies aimed at reducing the risks of adverse health outcomes related to frailty, respectively complexity?

## Method

### Study design

The fraXity study relies on a case-control longitudinal design with three measurement occasions (baseline, follow-up 1 and follow-up 2), each separated by a six-month interval.

### Setting

The study will be conducted in Canton Geneva, Switzerland, from September 1, 2018, to August 31, 2020, for an overall duration of 24 months. Recruitment began on October 1, 2018, and is expected to end by April 30, 2019. Data collection started October 30, 2018, and is expected to end by May 15, 2020, covering baseline assessment (expected from October 30, 2018, to May 15, 2019), follow-up 1 (expected from April 30, 2019, to November 15, 2019), and follow-up 2 (expected from October 30, 2019, to May 15, 2020).

### Participants

**Fig. 1:**
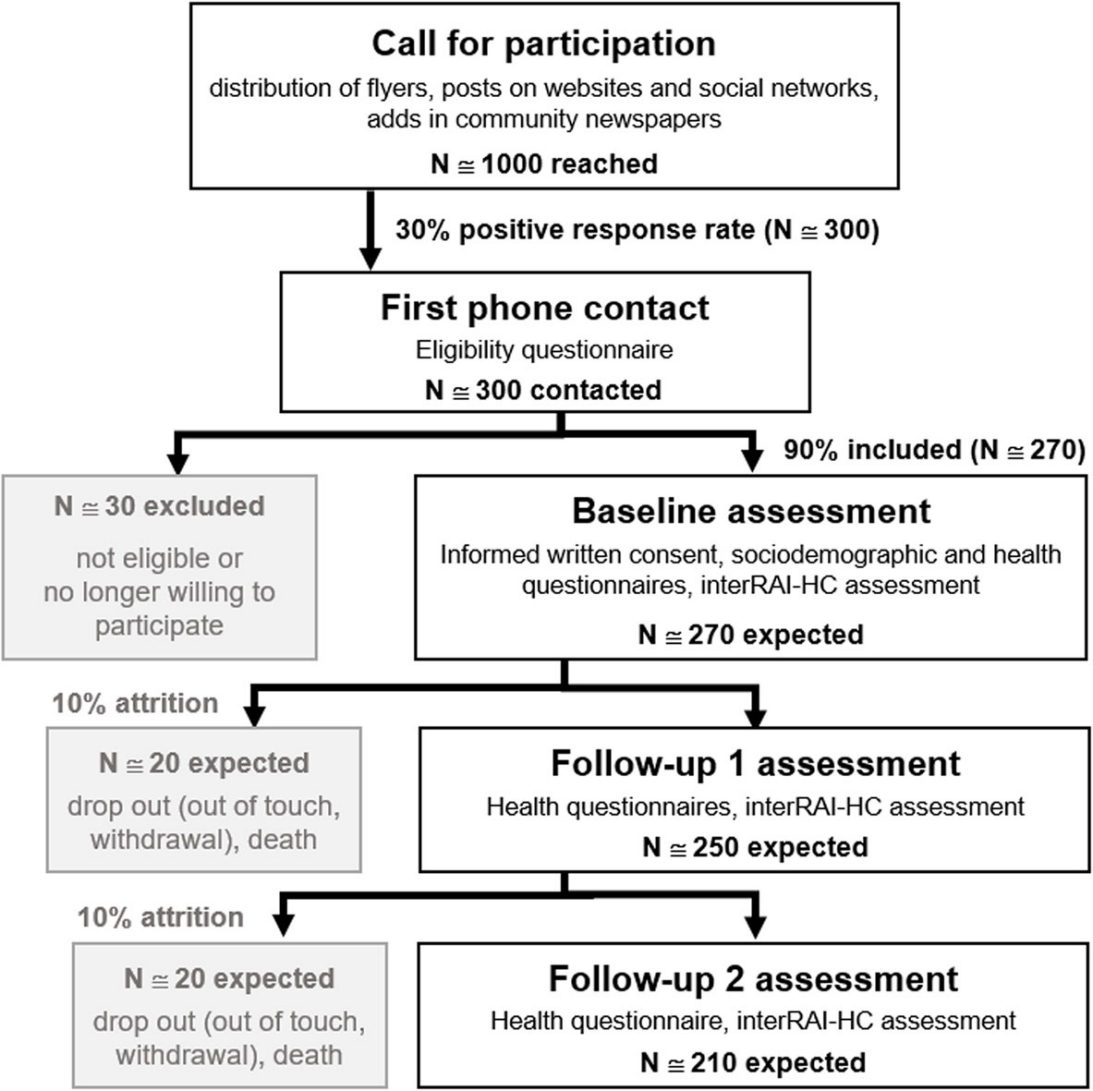
Flow chart summarizing the general procedure of the fraXity study

The target population is individuals aged 65 or older living in the community, receiving or not, professional home services. Participants will be divided among three samples based on an adverse health outcome risk stratification approach [40]. The criteria used to assign participants to each of the groups sampled is the use of formal home services, viewed as a proxy variable of the degree of vulnerability and risk of adverse outcomes. In the “control” group (lower risk, *N* = 70), participants are free of formal homecare or assistance. In the “assistance” group (medium risk, case 1, *N* = 70), participants are free of formal care but benefit from home assistance at least once a week. The types of services considered as formal assistance are help for household, shopping, meal preparation, transportation, administration and the use of meal delivery services. In the “care” group (higher risk, case 2, N = 70), participants receive formal homecare at least once a week, eventually in addition to formal assistance. Care is a service that the Swiss health insurance system recognizes and that a nurse, a nurse assistant or another health professional provides. Sampling will involve the use of a non-probabilistic convenience method; volunteers meeting the eligibility criteria will be enrolled in the study on a first-come, first-served basis. Eligible participants include men and women aged 65 or older, living in the community. For all participants, the inclusion criteria are as follows: to live in a private dwelling in the canton of Geneva, Switzerland; to be able to hold a meaningful conversation in French; and to be oriented in time and space. The two latter ones are established via the nurses’ clinical judgement at first phone contact, guided by target questions in the eligibility questionnaire. Exclusion criteria are as follows: to live in a long-term facility or in a nursing home; not living in the canton of Geneva, Switzerland; not fluent in French; disoriented in time and space; being subject of trusteeship. Eligibility is determined at first contact (see Fig. 1).

### Procedure

The general procedure of the fraXity study is summarized in Fig. 1. Recruitment will start with the sending out of calls for participation using the distribution of flyers on various occasions (e.g. community events, public conferences, senior citizens’ association meetings), posts on intuitional websites and ads in local community journals. Efforts will be made to cover all municipalities of the canton of Geneva so as to enhance the representativity of the sample by reaching individuals living in various socioeconomic neighborhoods and environments (e.g. urban vs. rural). Home service professionals from private and public institutions operating in the area will also distribute flyers to increase opportunities to reach individuals who benefit from home services (assistance or care). Candidates volunteering to participate manifest their interest by contacting the research team by phone, by regular mail or by e-mail. Subsequently, the fraXity staff organizes a first phone appointment (first contact) for eligibility assessment.

The aim of the first contact will be to verify eligibility criteria, assign temporarily each participant to a group, arrange a first appointment for assessment upon verbal agreement for participation and, if needed, provide additional information on the study. A dedicated eligibility questionnaire (EQ) serves to guide the interview by reviewing all of the eligibility criteria and by documenting the types of care and assistance from which the respondent eventually benefits.

At the first meeting, the nurse in charge of the data collection will begin by ensuring that the participant provides his/her written informed consent for participation prior to any data collection. Participants can enroll in the study for a 12-month period, during which they receive three assessments: a baseline and two follow-ups. Assessments will take place at the participants’ homes unless a person prefers a different place. As a rule, the same nurse will follow the participant throughout the study. In the event of non-returns across measurement occasions, the nurses will document the reasons for such: withdrawal, out of sight, death. Whenever possible, the nurses will also record the motives for withdrawals. Deaths are to be documented from administrative records and/or information that the relatives provide.

### Data sources and measurement

Nurses previously trained to complete comprehensive geriatric assessments with the interRAI-HC and additional instruments will collect the data. On each measurement occasion, participants will receive the interRAI-HC, which provides the outcomes used to derive the frailty (FI) and complexity (CI) indices, and a life history calendar (LHC), which provides the outcomes used to assess the predictive validity of FI and CI.

The interRAI-HC, Canadian French version 9.1 (243 items, 90 min [38]) is a standardized comprehensive geriatric assessment that belongs to a suite of instruments designed for standardized assessment across settings [41] and for consistent information sharing [42]. Version 9.1 of the Canadian French interRAI-HC entails a minimal dataset (MDS) covering the following domains: administrative information (A) and living conditions (B), cognition (C), sensory abilities (D), health-related behavior (E) and social behavior (F), activities of daily living (ADL, G), continence (H), medical diagnoses (I), falls, physical abilities, physical symptoms and pain (J), nutrition (K), skin and feet problems (L), medication (M), ongoing therapies and formal care (N), advanced care instructions and legal representativity (O), informal care (P), living environment (Q), observed change in ADL (R) and record information (S). As previously demonstrated [28–32], the quality and nature of the information collected with the interRAI-HC will be rich enough to derive a variety of additional clinical scores and indices.

The life history calendar (LHC, 15 min [43]). The LHC is a retrospective method used to gather reasonably valid information on past events. The LHC allows for the fine-grained time-to-event recoding of events, and it appears to be well suited to documenting adverse outcomes. As designed for the fraXity study, the tool covers the 6 months preceding each interview. It will assess the history of falls, emergency admissions, hospitalizations and lengths of stays, physician visits and life-striking events (e.g. separation, relocation, death of kin, medical diagnosis).

Additional sociodemographic and health questionnaires will be used to document population characteristics; the choice of the instruments will follow the recommendations of the International Consortium for Health Outcomes Measurement (ICHOM) [44, 45].

The EuroQoL EQ-5D-3 L (six items, 5 min [46]) is used to evaluate self-perceived health-related quality of life.

The Mini Nutritional Assessment–Short Form (MNA-SF, seven items, 5 min [47]) is a standardized scale designed to evaluate the nutritional statuses of older adults.

The Montreal Cognitive Assessment (MoCA, 30 items, 20 min [48]) is used to evaluate global cognitive functioning.

Global health status will be assessed by means of an ad hoc questionnaire serving to document self-reported additional health outcomes that include visual and auditory difficulties, frailty status, fear of falling, pain, current pathologies, and alcohol and tobacco consumption. The questionnaire will serve to document the sample characteristics and complements the heath-related data collected with the interRAI-HC with external measures.

Sociodemographic status will be assessed by means of an ad hoc questionnaire that serves to document basic sociodemographic outcomes, including age, sex, educational and professional attainment, living arrangements, living conditions, social participation and caregiving, self-perceived isolation and loneliness, and overall satisfaction with life. The questionnaire will serve to document the sample characteristics and complements the sociodemographic data collected with the interRAI-HC with external measures.

At the end of each assessment, the COMID [37] will be used to assess the multidimensional complexity of the case/situation. It will serve as an external measure of complexity, which serves to assess the convergent validity of the CI derived from the interRAI-HC assessment.

### Primary outcome measures

The FI and the CI derived from the baseline interRAI-HC assessments will be the main primary outcome measures. These scale variables will hold a value ranging from 0 to 1.00, computed as the sum of items recorded with the interRAI-HC MDS divided by the number of items considered. Changes in FI and CI values across occasions will further be considered as additional primary outcomes. Changes will be expressed as a proportion, with the index value at baseline being the denominator and the observed index value at follow-up being the numerator.

### Secondary outcome measures

The events recoded by means of the LHC will the secondary outcome measures. They include the number of falls, hospitalization, physician visits, emergency admissions and deaths. They will be used to estimate the predictive validity of the FI and CI.

### Bias

Information bias will be addressed through the double-checking of the criteria used for group assignment, which are first identified based on information collected with the EQ and subsequently adjusted based on data collected at baseline with interRAI-HC. Furthermore, information bias will be addressed via statistically testing group differences on potential confounding variables, such as age, sex, education and living arrangement. Should group differences be significant (*p* < .05) on a given variable, the variable will be used as a covariate in subsequent analyses so as to control for its confounding effect. Selection bias, potentially enhanced by the convenient sampling method, is addressed at recruitment. Calls for participation will be sent out in all municipalities of the canton of Geneva to enhance the representativity of socioeconomic neighborhoods and environments (e.g. urban vs. rural). Furthermore, both public and private home service providers will relay calls for participation among their clients to enhance the representativity of care and service recipients. Interviewer bias, which challenges the quality of the data collected, will be addressed by providing specific training to the nurses in charge of data collection and prior field work. Training will focus on validity (appropriate knowledge of what each instrument actually measures), reliability (appropriate use of homogenous instructions in standardized questionnaires) and quality of data (avoiding missing values). Practice sessions were organized to ensure that the nurses properly master the measurement instruments prior to data collection. Finally, measurement bias will be addressed by using, whenever possible, standardized instruments with documented validity and/or reliability.

### Study size

At the end of the study, the target sample size will be 70 participants in each group, for a total of 210 participants. This sample size was determined using G*Power [49] applied to a multivariate analysis of variance (MANOVA) design assessing within-between interactions for three groups and three repeated measures, for an expected effect size of f(V) = .20, an α error probability of .05 and power (1-β error probability) of .90. The results provided a critical overall size of *N* = 195, which was raised to *N* = 210 so as to reach an equivalent sample size of *N* = 70 in each group. Provided the expected size at the end of the study, an estimated *N* = 325 initial contacts for each group was determined, using an initial response rate of 30% (*N* = 97 positive responses), a subsequent eligibility rate of 90% (*N* = 87 initial assessments) and a 10% attrition rate between measurement occasions (*N* = 78 at 6 months and N = 70 at 12 months).

### Statistical methods

Specific analyses are planned for assessing each objective of the study. In all cases, descriptive and inferential statistics will be used. A priori, the threshold for rejecting the null hypothesis will be *p* < .05, but more conservative thresholds of *p* < .01 or *p* < .001 could be applied if a) covariates are required in the analyses, or b) the sample size is smaller than expected. Additionally, for all analyses conducted using regression models, the 95% confidence intervals (95% CI) of estimated coefficients will be used, informing the effect size and the range of expected values in the population. The 95% CI will be used to interpret the results above and beyond the standard α error probability.

### Deriving valid and reliable indices of frailty and complexity

The derivation of the frailty index (FI) will be done according to the methodology that Searle et al. [39] proposed and that the research group previously applied [32]. The methodology will be used as a scaffold to derive the CI. Descriptive analyses will be conducted to characterize the distributions of the FI and the CI. The reliability of the FI and CI will be estimated using test-retest reliability procedures.

### Assessing the predictive validity of the FI and CI

FI and CI computed at baseline will be used as predictors of health outcomes documented with the LHC at follow-ups. Survival models, adapted to single (e.g., death, diagnosis) or multiple failure (e.g., hospitalizations, falls) sequences [50] will be used. Data will be aligned on first assessment (time-to-event procedure). These analyses will determine the probability of adverse events over time as a function of the FI, respectively CI, initial scores.

### Comparing groups with various risks of adverse outcomes

Preliminary analyses will be conducted to assess potential preexisting group differences on baseline sociodemographic and health variables, as the ICHOM recommends [44, 45]. The analyses will be conducted by means of one-way ANOVAs, chi-square tests and Kruskal-Wallis tests, respectively, for continuous, nominal and ordinal dependent variables. Variables demonstrating significant group differences at *p* < .05 will be subsequently used as covariates. Group comparisons on the FI and CI scores will be assessed at baseline using separate linear regressions, with group as predictors (control group as reference) and FI and CI as outcomes. Regression modeling will also be used at the end of data collection to estimate group differences on the evolution of frailty and complexity. The models will include the main effect of group (between subjects) and measurement occasion (within subject), as well as the two-way interaction (Group × Occasion). Finally, to assess group differences in terms of the risk of adverse outcomes, survival analyses will be conducted by including group as a covariate in the analysis design.

### Handling of missing data

A 10% attrition rate across measurement occasions is planned. Missing data due to dropouts will not be replaced. As far as possible, reasons for attrition will be documented, and dropouts will be compared with returning participants. Regarding missing data due to nonresponses during assessments, special care will be taken to avoid them during data collection, with the goal being to remain below 5% missing data over the entire sample. Subsisting missing values will not be replaced or imputed.

## Discussion

The expected outcomes of the fraXity study are a) reliable computation algorithms for frailty and complexity scores derived from the interRAI-HC, and b) clinical assessment protocols for use by homecare nurses, with specific recommendations for target subgroups of the older population and adapted care strategies designed to prevent undesirable health events and overwhelming health expenditures. By providing homecare nurses with frailty and complexity screening tools, as well as recommendations for use, the fraXity study should outfit key actors of the health system with means of enhancing their part in a collective endeavor targeting the best care and quality of life for aged citizens.

### Study status

The protocol version is 2.0. It was registered at ClinicalTrials.gov on March 20, 2019, with the identification number of NCT03883425.

At the time of the first submission of the manuscript, the study is ongoing. The recruitment of participants began on October 30, 2018, and is expected to end by May 15, 2019.

## Data Availability

The datasets generated (coded, free of personal information), used and analyzed during the fraXity study will be deposited at the end of the study at DARIS/FORS (http://forscenter.ch) for data sharing and reuse purposes. FORS/DARIS comply with the FAIR (findable, acceptable, interoperable, re-usable) principles.

## Abbreviations

ADL: Activities of daily living
CI: Complexity index
COMID: Multidimensional complexity assessment instrument
EFT: Equivalent full time
EQ: Eligibility questionnaire
EQ-5D-3 L: Thee-level version of the EuroQol questionnaire with five dimensions
FI: Frailty index
HES-SO: University of Applied Sciences and Arts Western Switzerland (Haute École Spécialisée de Suisse Occidentale)
ICHOM: International Consortium for Health Outcomes Measurement
imad: Geneva institution for homecare and assistance (institution genevoise de maintien à domicile)
LHC: Life history calendar
MDS: Minimal data set
MNA-SF: Mini Nutritional Assessment – Short Form
MoCA: Montreal Cognitive Assessment
RAI-HC: Resident Assessment Instrument – Home Care
